# Effects of Religious Priming Concepts on Prosocial Behavior Towards Ingroup and Outgroup

**DOI:** 10.5964/ejop.v12i4.1170

**Published:** 2016-11-18

**Authors:** Jame Bryan L. Batara, Pamela S. Franco, Mequia Angelo M. Quiachon, Dianelle Rose M. Sembrero

**Affiliations:** a Department of Psychology, University of San Carlos, Cebu City, Philippines; EJOP Editor; Aalborg University, Aalborg, Denmark

**Keywords:** religion, priming, prosocial behavior, ingroup and outgroup, religious primes

## Abstract

Several studies show that there is a connection between religion and prosociality (e.g., Saroglou, 2013). To investigate whether there is a causal relationship between these two variables, a growing number of scholars employed priming religious concepts and measure its influence on prosocial behavior (e.g., Pichon, Boccato, & Saroglou, 2007). In the recent development of religious priming, Ritter and Preston (2013) argued that different primes (agent prime, spiritual/abstract prime, and institutional prime) may also have varying influence on prosocial behavior specifically helping an ingroup or an outgroup target. With this in mind, a 2 (social categorization of the target of help) by 3 (agent prime, institutional prime, spiritual prime) experiment was conducted to directly investigate this hypothesis. Results suggest that priming religious concepts especially the spiritual prime can increase prosocial behaviors. However, no significant effect was found on the social categorization which implies that Filipino participants elicit prosocial behavior regardless of the social categorization (be it ingroup or outgroup) of the target of help. The present study’s findings contribute to further the literature on religious priming and its influence on prosocial behavior.

## Introduction

Helping as a social behavior is influenced by different factors. A growing bulk of studies, both correlational and experimental, point to the influencing factor of religion towards helping. However, experimental investigations through priming religious contexts and concepts have led to mixed results (for recent review, see Shariff, Willard, Andersen, & Norenzayan, 2016). In his review on the studies of religion and helping (Saroglou, 2013), religiosity has been more salient in helping the ingroup. For example, there was unwillingness to help illegal immigrants among those with orthodox religious beliefs (Pichon & Saroglou, 2009) and preference to help an ingroup family (than outgroup) move in (Preston & Ritter, 2013).

It has been suggested that the priming concepts employed in the experiments investigating the influence of religion to prosocial behavior need to be clarified so as to arrive to more meaningful results. Ritter and Preston (2013) suggested that the three different religion-related primes (agent, spiritual, institutional) may lead to different levels of prosocial behavior. With this in mind, the present study looked into the influence of the three priming concepts on prosocial behavior towards ingroup and outgroup targets. It is the aim of this study to provide a clearer picture of the influence of religion-related primes on prosocial behavior.

Religion is social in nature (McCullough & Willoughby, 2009). This clearly indicates that engaging in religion also has social repercussions. Human beings make connections to which they believe as divine, spiritual, sacred, and holy (Mehanna, 2003). The basic element of most religions is to worship and make deeper connection with a supreme being (Helble, 2006). Hill and colleagues (2000) conceptualized religion as the psychological processes in relation to the recognition of the sacred and the engagement in religious practices in the presence of a community of believers. The same authors indicated that spirituality is related to psychological process in search for the sacred that is done in one’s private sphere. In light of this, the present study focuses on religious priming that can be reflective of spirituality and religion.

It has long been argued and is empirically supported that religion has an impact to the different areas of an individual’s life (Oviedo, 2015). One of these areas is its influence towards prosocial behavior (Saroglou, 2013). Prosocial behavior is an action that serves to benefit an individual or group (Eisenberg, Fabes, & Spinrad, 2007). Several correlational studies indicate that religiosity increases willingness to volunteer (Park & Smith, 2000), and that religious people are participative in activities for charity in both secular and religious contexts (Lam, 2002). Even among those low-income religious individuals, acts of charity are still observed (Myers, 2012).

Experimental investigations also indicate that religion influences prosocial behavior. One of the most employed methods for these experimental investigations is priming. Priming involves activating related conceptual representations in an individual’s memory which can lead to thinking and behaviors in line with such activated representations (Bargh, Chert, & Burrows, 1996). For example, when primed with “God”, participants provided more monetary allocations in an anonymous economic game (Shariff & Norenzayan, 2007). University students who were primed with a watching God through listening to an English song and then translating it into one’s language offered more time for volunteer work compared to those who did not listen to and did not translate a religious song (Batara, 2016). Word primes related to religion also increased intention to help (Pichon, Boccato, & Saroglou, 2007), willingness to volunteer (Sasaki et al., 2013), and honesty (Randolph-Seng & Nielsen, 2007). Finally, contexts related to church and religion increase willingness to help and actual helping behavior (Ahmed & Salas, 2013; Pichon & Saroglou, 2009; Ruffle & Sosis, 2010).

These interesting religious priming studies indicate that activating thoughts about one’s religion can increase prosocial behavior. However, Saroglou (2013) suggested that religion has been found to be more salient in helping an ingroup than an outgroup, a phenomenon he called “minimal prosociality”. Other studies found that religious primes reduced prosocial thoughts and behaviors. For example, Ginges, Hansen, and Norenzayan (2009) found in their cognitive priming experiment that those primed with frequency of synagogue attendance demonstrated more support to suicide attacks compared to those primed with frequency of praying to God. Another study found that students primed with religion-related words (e.g., church, bible, sermon) showed slightly higher racial prejudice compared to those primed with neutral words (e.g., shirt, butter, switch) (Johnson, Rowatt, & LaBouff, 2010). It is noticeable in these two aforementioned studies (e.g., Ginges et al., 2009; Johnson et al., 2010) that the primes being used somehow pointed to the institutional nature of religion (e.g., church and synagogue attendance) which may have some varying influence to prosocial behaviors compared to those primed with different religion-related primes. With this, Ritter and Preston (2013) suggested that the varying religion-related primes previous researchers used may have also activated varying religion-related prosocial behaviors in the participants. These different religious primes activating different psychological functions could have been one of the reasons for these mixed results.

The three religion-related primes are religious agents (e.g., God, angel), spiritual/abstract prime (e.g., divine, sacred), and institutional/concrete prime (e.g., ritual, scripture). Agent primes were facilitative of prosocial behavior towards both ingroup and outgroup (Ritter & Preston, 2013). Agent primes are concepts that depict an entity (or entities) known to have divine qualities. Some identified religious agents are as follows: God, saint, prophet, and angel (Ritter & Preston, 2013). Previous empirical evidence showed that priming these religious agents can increase certain behaviors toward two targets, the ingroup and outgroup. For example, priming participants with the word “God” increased likelihood of cooperation with an outgroup compared to those primed with “religion” (Preston & Ritter, 2013).

Another religious concept that can be manipulated to increase prosocial behavior is the spiritual/abstract prime. Spiritual/abstract primes are priming concepts that relate to the connection of an individual with the sacred such us faith, miracle, revelation and heaven (Ritter & Preston, 2013). Religious words such as faith and beliefs increase prosocial behavior (Pichon, Boccato, & Saroglou, 2007).

Lastly, institutional prime relates to the institutional aspect of religion. Graham and Haidt (2010) identified institutional prime as an influence of the culture that constitute people into moral communities. People who pray inside the church created a sense of kinship towards their religious circle and have resulted to eliciting parochial altruism (Choi & Bowles, 2007). Exposing participants to a picture of a person in need in front of a church resulted to more generosity; however, this effect was limited to proximal in-group (homeless) and was not extended to distant, outgroup-like targets (i.e., illegal immigrants) (Pichon & Saroglou, 2009).

Although a growing number of studies used priming methodology to study the causal connection of religion and prosociality, there is still a need to investigate whether the three kinds of religious primes have varying influence to prosocial behavior between ingroup and outgroup targets.

## Methods

### Research Design

A 2 (social categorization of the target of help) by 3 (agent prime, institutional prime, spiritual prime) experimental design was used for this study. For each condition, a priming concept (agent, institutional, and spiritual) and the target of help (ingroup and outgroup) were shown in a video clip. The present study has undergone ethics review and has been given clearance under ethical considerations by the University of San Carlos Institutional Ethics Review Committee. Complete experimental protocol is available upon request.

### Participants

One hundred seventy three participants completed the experiment (97 males and 76 females). The age range was 16 – 21 years old (*M* = 18.6, *SD* = 11.7). Participants were all undergraduates of the University of San Carlos – Talamban Campus. There were six conditions in the experiment. These conditions were abstract-ingroup (*n* = 27; 17 males, 10 females), abstract-outgroup (*n* = 29; 20 males, 9 females), agent-ingroup (*n* = 30; 12 males, 18 females), agent-outgroup (*n* = 30; 17 males, 13 females), institutional-ingroup (*n* = 30; 13 males, 17 females), and institutional-outgroup (*n* = 27; 18 males, 9 females).

### Measures

The researchers used video clips and coupons. Priming concepts and the social categorization of the target of help were shown in the video. The coupons measured the level of prosociality. The more coupons the participants asked from the experimenters, the higher the level of prosociality. Further details are stipulated in the succeeding paragraphs.

In the video, a person who is the beneficiary of the organization appeared and was interviewed. Religious contexts such as image of God (agent prime), dove (abstract prime), and church (institutional prime) appeared in the video according to the experimental condition the participants was randomly assigned into. The said beneficiary introduced himself as a Carolinian (student from the same university) for the ingroup condition and as a student (no specific university coming from) for the outgroup condition. Crisp and Beck (2005) demonstrated that the ingroup and outgroup categorization among university students is present. However, indicating the overlap of the ingroup and outgroup university students may decrease the intergroup bias between the two. In the present study, no further introduction of the university-related similarities (or overlap) of the ingroup and outgroup students except the idea that they need help in terms of education support. Thus, the present study’s procedure was able to produce an ingroup-outgroup impression of the student in help. During the interview shown on the video clip, the beneficiary stated a line with the priming concept (depending on the condition) as also part of the manipulation of prime. A quote related with the priming concept appeared before the video ends. Prosocial behavior was measured through the number of coupons the participants were willing to sell to help the beneficiary.

In this connection, a pilot experiment was conducted to ensure that the experimental procedure and the necessary experiment materials are fit and ready for the actual experiment. The results of the pilot experiment led to improvement of the experiment methodology in general (e.g., improved procedure, improved video clips for priming manipulation). The concepts (dove for spiritual prime, God for agent prime, church for institutional prime), as empirically validated by Ritter and Preston (2013), were representative of religious primes. In the case of the present study, these concepts were presented in a video clip.

### Procedure

The researchers gathered participants by making an event through social networking sites and any other media of communication. The said event was a cover story to minimize suspicion from the participants. The said orientation was held in an available classroom inside the university. Researchers also wore shirts with an “Abuno Foundation” as uniforms and as part of the cover story.

It has to be noted that deception was employed to conceal the true nature of the activity. The deception was about misleading the participants by telling them that Abuno Foundation is a new organization in the university. The deception is employed so as to elicit real-life behavior from the participants. Batson (2002) suggested that it may be preferred to measure actual behavior from a seemingly actual event so that it can represent a more real-life prosocial behavior. The deception only entailed minimal risk to the participants and were given proper debriefing after the entire experiment.

Participants were randomly assigned into conditions. Each participant underwent only one condition. Ostensibly introduced as an orientation about the university’s newly registered organization which is “Abuno Foundation”, the experimenters (Abuno Foundation representatives) waited for the number of participants to be at least five or maximum of ten. Upon reaching this number, experimenters randomly assign (through simple draw by lots) this group of participants into one experimental condition. The participants were not allowed to sit near their friends to avoid comparing of responses and filled-up information. The participants were given an informed consent before the orientation started.

Experimenters introduced themselves and told the participants to watch the video. There were six (6) videos which were shown in a classroom with a projector. One video was shown per condition. Video A contained an agent prime and the beneficiary presented himself as a Carolinian (ingroup). Video B contained the same prime with Video A but the beneficiary presented himself as a college student (outgroup). The primes used for Video A and B were the image of God as a background and the word “God” was mentioned by the beneficiary and was shown as an ending quotation. Video C contained spiritual/abstract prime and the beneficiary presented himself as a Carolinian (ingroup). Video D contained the same prime with Video C but the beneficiary presented himself as a college student (outgroup). The primes used for videos C and D were the image of the dove as a background and the word “faith” was mentioned by the beneficiary and was shown as an ending quotation. Video E contained an institutional prime and the beneficiary presented himself as a Carolinian (ingroup). Video F contained the same prime with video E but the beneficiary presented himself as a college student (outgroup). The primes used for Video E and F were the church as the setting and the words “*simbahan*” (church) and “*mag-ampo*” (praying) were mentioned by the beneficiary and were shown as an ending quotation. After the video was shown, the experimenter announced about selling the coupons that could help the student in need. Another experimenter led the participants to the corner where the coupons were displayed. It was up to the participants how many coupons they pledged to sell. An experimenter outside debriefed the participants at the end of the experiment.

## Results

The present study looked into the influence of the three priming concepts on prosocial behavior towards ingroup and outgroup targets. Although the age of the participants was relatively homogeneous, it is noticed that the number of males and females in each experimental condition was not evenly distributed. Thus, the number of males and females were controlled in the main analysis. Controlling for gender category, *F*(1, 166) = 0.018, *p* = .893, Two-Way Analysis of Covariance (see Table 1) shows no interaction between priming concepts and social category, *F*(2, 166) = 1.432, *p* = .242, as well as no main effect in social category, *F*(1, 166) = 1.683, *p* = .196.

**Table 1:** Two-Way Analysis of Covariance Summary of Results

Source	*SS*	*df*	*MS*	*F*
**Gender (covariate)**	0.397	1	0.397	0.018^ns^
Main Effects				
Priming Concept	224.830	2	112.415	5.146**
Category of Target	36.765	1	36.765	1.683^ns^
**Interaction Effect**	62.548	2	31.274	1.432^ns^
**Within Groups**	3626.107	166	21.844	
**Total**	3959.630	172		

**p < .01. ^ns^not significant.

Priming concepts showed significant main effect, *F*(2, 166) = 5.146, *p* = .007. The effect size was moderate (partial eta squared = .058). Post-hoc comparison using Tukey’s HSD indicated that spiritual prime (*M* = 7.6, *SD* = 6.1) elicited the most prosocial behaviors measured by the number of coupons the respondents were willing to distribute. There was no significant difference between agent prime (*M* = 5.1, *SD* = 3.7) and institutional prime (*M* = 5.2, *SD* = 3.9). See Table 2 below for the mean and standard deviation of all conditions.

**Table 2:** Mean and Standard Deviation of the 6 Experimental Conditions

Prime	*M*	*SD*
Agent (Total)	5.08	3.72
Ingroup	5.47	4.44
Outgroup	4.70	2.85
Institutional (Total)	5.21	3.95
Ingroup	4.40	2.90
Outgroup	6.11	4.76
Abstract/Spiritual (total)	7.64	6.08
Ingroup	6.70	5.61
Outgroup	8.52	6.46
Social Category		
Ingroup (total)	5.48	4.46
Outgroup (total)	6.43	5.10

In general, spiritual/abstract prime elicited the most prosocial behavior. Interestingly, the social category of the target of help did not matter in influencing prosocial behavior; that is, no matter who the target of help is, the participant still engaged in prosocial behavior.

## General Discussion

Several studies have used priming methodologies to investigate the effect of religious concepts to prosocial behavior; however, there is a need to directly compare the three priming concepts namely agent prime, spiritual/abstract prime, and institutional prime and its influence to ingroup and outgroup targets of help. The goal of the present study was to investigate the effects of priming concepts and social categorization towards prosocial behavior.

The present study found that priming concepts, specifically spiritual primes can increase prosocial behavior. It can be construed that religious words seem to reflect either concrete objects or abstract concepts. In line with the construal level theory, abstract thinking and concrete thinking have different effects on the people’s cognition and behavior (Trope & Liberman, 2010). Ritter and Preston (2013) suggested that spiritual prime activates abstract thinking and such thinking is related to universality in treating and dealing with people. For example, abstract thinking led Christians to exhibit less prejudice towards outgroup (Luguri, Napier, & Dovidio, 2012). Thus, among the three primes, spiritual prime elicited the most prosocial behavior because it appears to activate abstract thinking which in turn leads to nonbiased and increased engagement in prosocial behavior.

In a recent review on religious prosociality, it has been consistent that religiosity is more associated with helping the ingroup (Saroglou, 2013). However, in the present study, no distinction was found in prosocial behavior between ingroup and outgroup targets. Interestingly, this reflects the collectivist nature of the Filipino participants. Same findings were found among Filipino respondents’ willingness to help regardless of the social categorization of the target (Batara, 2015). Filipinos tend to give more importance in maintaining harmonious relationships than in categorizing people as ingroup/outgroup (del Prado et al., 2007). This may be the reason why social category of the target of help has no effect on prosocial behavior.

Regardless of who is in need, Filipino participants were still willing to help the beneficiary. *Kapwa* theory indicates that Filipinos treat both close and distant others as part of their shared inner self and this somehow reflects in interacting with others (Enriquez, 1992). *Kapwa* denotes a Filipino’s awareness and sense of shared identity with others and it has been practiced early in the family which then extends beyond one’s family as one grows and socializes with others (Enriquez, 1978). Indeed, it is not about maintaining smooth interpersonal relationship alone but Filipinos are most concerned with *pakikipagkapwa* or treating others as fellow (Clemente et al., 2008). Thus, the non-distinction of prosocial behavior towards ingroup and outgroup reflects the Filipinos’ sense of oneness with co-Filipinos. Direct investigation of the sense of maintaining harmonious relationship as a possible mediating factor for the link between religion and prosociality may provide further insights.

The present study was able to address the need in directly comparing the three priming religious concepts and its influence to ingroup and outgroup targets of help. However, several limitations have to be noted. The experimental setting used in the present study was in a classroom setting providing limited space, leading to interaction towards the participants creating suggestive changes or influencing other participants on how many coupons to get. The researchers were not also able to measure the religiosity of the participants which can also be an important influencing factor in the study. Participants were college students with age ranging from 16 to 21 years old. Extending the age range of participants may strengthen the generalizability of the results.

The present study showed that one can increase their prosocial behavior by just being exposed to religious contexts and concepts. Priming may be temporary but still it can increase one’s prosocial behavior in certain situations. People, particularly Filipinos, tend to help those in need regardless of the social categorization.

### Conclusion

Helping other people is one of the central values of religion (Batson, 1990). One of the purposes of many religions is to unify people in helping the community (Graham & Haidt, 2010). With this in mind, the present study provides valuable insights into the link of religious priming, social categorization and prosociality. In summary, people who are primed spiritually had the sense of helping others more than those who were exposed to other known religious primes (agent and institutional). Depending on the cultural context, there may be some differences in helping an ingroup or outgroup. In the case of Filipinos, no distinction was found. The present study helps to clarify the varying influence of religious priming concepts on prosocial behavior towards ingroup and outgroup members.
